# *Eucommiae cortex* Comprehensive Phytochemical Analysis Connected with Its In Vitro Anti-Inflammatory Activity in Human Immune Cells

**DOI:** 10.3390/molecules30061364

**Published:** 2025-03-18

**Authors:** Małgorzata Kołtun-Jasion, Marta Katarzyna Dudek, Anna Karolina Kiss

**Affiliations:** 1 Department of Pharmaceutical Biology, Faculty of Pharmacy, Medical University of Warsaw, Banacha 1, 02-097 Warsaw, Poland; 2 Centre of Molecular and Macromolecular Studies, Polish Academy of Sciences, Sienkiewicza H. 112, 90-001 Łódź, Poland; marta.dudek@cbmm.lodz.pl

**Keywords:** *Eucommia ulmoides*, lignans, iridoids, monocytes, macrophages, neutrophils, inflammation

## Abstract

*Eucommia ulmoides* Oliv., commonly known as “plant gold”, is a species of the Eucommiaceae family, native to East Asia and widely utilized in medicine, food, and the chemical industry. In Traditional Chinese Medicine, the bark of *E. ulmoides* plays a special role, used to nourish the liver and kidneys and to strengthen tendons and bones. Due to its extensive pharmacological profile, including anti-inflammatory, antioxidant, hypoglycemic, hypotensive, and cardio- and neuroprotective effects, there has been growing interest in elucidating the molecular mechanisms underlying its biological effects. However, many of these mechanisms remain poorly understood to date. This study analyzed the phytochemical composition of *E. ulmoides* bark infusions and tinctures and their dominant compounds using the HPLC-DAD-MS/MS method, and evaluated their anti-inflammatory effects in human immune cell models. The analysis identified lignans, iridoids, and caffeic acid derivatives as the dominant constituents of the tested samples. The extracts significantly inhibited the secretion of pro-inflammatory cytokines (TNF-α, IL-6, IL-8, MCP-1) in neutrophils, PBMC-derived monocytes/macrophages, and THP-1 cells. The results presented herein offer significant insights into the detailed phytochemical composition of *E. ulmoides* bark, and contribute to a deeper understanding of its anti-inflammatory mechanisms in human immune cells.

## 1. Introduction

*Eucommia ulmoides* Oliv. (*EU*) is the sole and representative species of the Eucommiaceae family, characterized as small trees with significant therapeutic and economic value [1]. With the expanding presence of this species in Europe, its bark, leaves, stems, flowers, and seeds have gained recognition for their analgesic, antimicrobial, antihypertensive, antihyperlipidemic, antihyperglycemic, insulin-sensitizing and antioxidant properties, in addition to their good safety profile. In Traditional Chinese Medicine (TCM), *Eucommiae cortex* (*EC*) is especially valued, with the Chinese Pharmacopoeia recommending its use for bone and tendon reinforcement and kidney-tonifying activity. Moreover, *EU* bark extract is an active component used for antihypertensive formulations [2,3,4]. Modern pharmacological research has highlighted the significant therapeutic potential of *EC* or its main constituents, demonstrating their efficacy in treating hyperglycemia [5], diabetes, obesity [6], renal dysfunctions [7,8], osteoporosis [9], epilepsy, and neurodegenerative diseases [10], thus leading to longevity and promoting human fertility.

A common factor linking most of the aforementioned disorders is the progressive inflammatory state, which evolves from a low-grade inflammation to a chronic condition that can persist for several months. One of the main topics discussed in the context of assessing the effectiveness and potential areas of application of plant materials is their impact on limiting the processes of ongoing inflammation. Furthermore, an increasing number of studies correlate the phytochemical composition of medicinal plant materials and their components with observed biological activities. The health-promoting properties of *EC* are largely attributed to the presence of lignans (such as pinoresinol, syringaresinol, medioresinol, olivil, and their derivatives), caffeic acid derivatives, as well as iridoids (including aucubin, geniposidic acid, and eucomoside B), which have been identified as key chemical groups in the most popular forms of *EC* use—self-made tea infusions and tinctures.

Reports on the chemical composition of *EC* extracts are incomplete. Although a substantial body of literature exists on the phytochemical properties of *Eucommia* leaves, stems, and seeds, relatively few studies have focused on the pharmacological properties of *Eucommiae* bark and its main compounds, particularly in the context of their effects on human immune cells, involved in the development of inflammatory states. As *E. ulmoides* leaves and buds are extensively used as food additives in Asia, it is of interest whether the consumption or medicinal use of its bark may also exert health-promoting effects. Furthermore, it is worth investigating whether *EC* could be incorporated as a component in the therapy and prevention of diseases, particularly those with an inflammatory basis.

The present study aims to evaluate the phytochemical compositions of two commonly utilized pharmaceutical preparations of *Eucommia ulmoides* bark: infusions and alcoholic extracts (tinctures). The study focused on comparing the phytochemical profiles and biological activities of different forms of plant-based pharmaceutical formulations, as well as isolating their dominant compounds. In particular, the anti-inflammatory and antioxidant properties of the examined extracts and isolated compounds were assessed in vitro using human leukocytes. This approach confirmed the distinct anti-inflammatory properties of *E. ulmoides* preparations, underscoring their significant therapeutic potential. Moreover, the research findings indicate that *Eucommia ulmoides* serve as a valuable source of compounds belonging to the lignan, irydoids and fenolic acids derivatives groups, which are potentially responsible for the observed anti-inflammatory effects of the raw material. The presented results may serve as a foundation for further studies aimed at confirming the significance of the *E. ulmoides* cortex in medicine and therapy.

## 2. Results

### 2.1. HPLC-DAD-MS/MS Analysis

**Figure 1:**
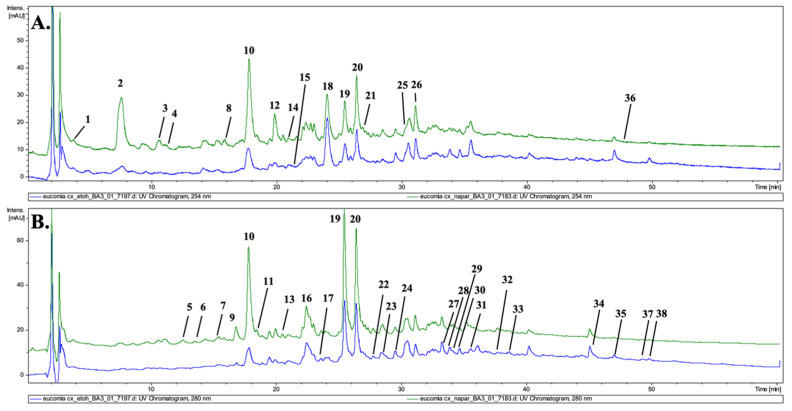
UHPLC-DAD-MS chromatogram of *Eucommiae cortex* infusion (green) and tincture (blue) recorded at 254 (**A**) and 280 nm (**B**).

The chromatographic analysis of the aqueous and alcoholic extracts led to the detection of 38 constituents (Figure 1, Table 1). Based on the UV–Vis maxima present in recorded spectra, the compounds were divided into three main groups of phytochemicals. Compounds **4**, **5**, **10**, **11** and **32** displayed maxima in the range between 300 and 325 nm and were classified as caffeic acid derivatives. Compounds **1**, **2**, **12**, **18** and **26**, with maxima at 225–230 nm, were preliminarily identified as iridoids. Additionally, compounds **9**, **14**, **16**, **17**, **19**–**24**, **27**–**29**, **33**, and **35**, with a characteristic maximum at 270–280 nm, classified to the lignans group (or their derivatives), were detected during the analysis.

Compounds **3**, **4**, **10**, **11** and **32** exhibited a primary pseudomolecular ion (or fragmentation pattern) in the negative MS spectrum at *m*/*z* 353.41 (C_16_H_17_O_9_) [caffeoyquinic acid–H]^−^ and also shared a similar MS^2^ product ion at *m*/*z* 191.05 (C_7_H_11_O_6_) [quinic acid–H]^−^. Compounds **4**, **10** and **11** were identified as isomeric forms of caffeoylquinic acids. The detailed examination of fragmentation pathways and the analysis of elution order on a C18 column enabled the identification of 3-*O*- and 5-*O*- and 4-*O*-caffeoylquinic acid, respectively [11].

Compound **5** (t_R_ = 12.6) exhibited a pseudomolecular ion at *m*/*z* 515.30 [M-H]^−^, analogous to compound **32**. The MS^2^ fragmentation revealed a signal at *m*/*z* 353.02, leading to the identification of the compounds as caffeoyl-*O*-(glucosyl)quinic acid and dicaffeoylquinic acid, respectively.

A total of five iridoids were identified in this study, including compounds **1**, **2**, **12**, **18** and **26**. Compound 1, with a precursor ion [M-H]^−^ at *m*/*z* 345.11 and a fragmentation pattern dominated by an ion at *m*/*z* 182.82, was identified as aucubin. Compound **2** (t_R_ = 7.7), exhibiting a pseudomolecular ion at *m*/*z* 373.21 and an MS^2^ fragmentation pattern at *m*/*z* 210.76 [M-H-Glc]^−^, was identified as geniposidic acid. Similarly, the pseudomolecular ion at *m*/*z* 225.10 [M-H]^−^, with a fragmentation profile at *m*/*z* 207.08 and 122.79, enabled the identification of genipin (**18** t_R_ = 24.1). Furthermore, the analysis enabled the identification of eucomoside B (compound **26**, *m*/*z* 520.29), an iridoid specific to *EC*, with fragmentation patterns at *m*/z 355.03, 233.83, and 190.75, consistent with previous reports [12,13].

The main class of secondary metabolites identified in the tested extracts were lignans, which predominantly occured in the analyzed raw material in the form of glucoside conjugates. Our analysis has allowed us to tentatively identify **15** compounds from this group. The HPLC-MS analysis of lignans revealed the presence of characteristic aglycone profiles and their glycosidic derivatives. These compounds include pinoresinol (*m*/*z* 357.29), syringaresinol (*m*/*z* 417.14), hydroxypinoresinol (*m*/*z* 373.19), and olivil (*m*/*z* 375.10).

The leading representative of lignans in *EC* was pinoresinol di-*O*-glucoside, identified as compounds **19** (t_R_ = 25.5) and **20** (t_R_ = 26.4), with *m*/*z* 681 [M-H]^−^. Its MS^2^ fragment ions at *m*/*z* 519.18 and *m*/*z* 357.29 were observed due to the loss of 1 and 2 glucose moieties, respectively. Further fragmentation corresponded to the next stages of cleavage of the tetrahydrofuran ring, confirming the presence of pinoresinol aglycone. The base peak at *m*/*z* 519 was attributed to two peaks: **27** (t_R_ = 33.3) and **28** (t_R_ = 33.9), which yielded very similar spectra in the MS^n^ analysis. Accordingly, both peaks were identified as isomeric forms of pinoresinol/ epipinoresinol glucoside, with a fragmentation pattern at *m*/*z* 357.34. According to a previous report by He et al., compound **33** (t_R_ = 38.6) with *m*/*z* 831.37 [M-H]^−^ was identified as pinoresinol vanillic acid ether diglucoside, while compound **36** was identified as its monoglucoside (*m*/z 699.24 [M-H]^−^) [14].

Hydroxypinoresinol-*O*-diglucoside (**16**, t_R_ = 22.3) and its diglucoside (**23**, t_R_ = 28.4) presented a characteristic pattern in its fragmentation profile. After the loss of hexose moiety from the pseudomolecular ion at *m*/*z* 535.14 [M-H]^−^, the aglycone signal at *m*/*z* 373.07 first underwent the characteristic cleavage of the tetrahydrofuran ring to produce the ion at *m*/z 343, followed by the cleavage of another tetrahydrofuran ring to yield *m*/*z* 313.31.

The next group of compounds from the lignan group were olivil derivatives. Compound **9** (t_R_ = 16.8) showed a pseudomolecular ion signal at *m*/*z* 699.22 [M-H]^−^. The cleavage of two glucose molecules gave fragmentation ions at *m*/z 537.17 and 375.10, respectively. This allowed the identification of olivil-di-*O*-glucoside. Similarly, compounds **14** (t_R_ = 21.0) and **17** (t_R_ = 22.8) were identified, based on the presence of one glucose molecule, as olivil-*O*-glucosides.

Compounds **22** and **29** presented aglycone moiety at *m*/*z* 417.14 in their fragmentation pattern. Both of them were assigned as different glucosides of the same aglycone. Compound **22** showed in its fragmentation spectrum the cleavage of two hexose units leading to syringaresinol residue at *m*/*z* 417. Thus, **22** (t_R_ = 27.7) was assigned as syringaresinol-di-*O*-glucoside (741.33 [M-H]^−^), and **29** (t_R_ = 34.3) as syringaresinol-*O*-glucoside (579.33 [M-H]^−^).

As regards other compounds (**3**, **6**, **7**, **8**, **13**, **25**, **30**, **31**, **34**, **35**, and **38**), it was impossible to identify them based on their key characteristic ions due to the lack of detail information about their shared structure.

**Table 1:** Retention time, UV, and MS/MS data of the compounds present in the tested extracts.

	Compound	UV (nm)	Rt (min)	[M-H]^−^ [M + COOH]^−^	Product Mass Peaks	Group	Infusion	Tincture	Ref.
1.	Aucubin	245	3.2	391.20 *	345.11 182.82	Irydoids	+	−	[12,15]
2.	Geniposidic acid	240	7.7	373.21	210.76 166.72 122.79	Irydoids	+	+	[12,15]
3.	Unknown	195	10.7	447.31	409.10 378.81 314.94 160.91	−	+	+	−
4.	3-*O*-caffeoylquinic acid	322	11.1	353.47	190.77	Caffeic acid derivatives	+	−	[12,15]
5.	Caffeoyl-*O*-(glucosyl) quinic acid	320	12.6	515.29	353.02 190.73	Caffeic acid derivatives	+	−	[12,14]
6.	Unknown	−	13.7	551.29	541.38 487.18 380.18 267.80	−	+	+	−
7.	Unknown	−	15.3	599.27 *	553.20 391.06 373.13 361.06	−	+	+	−
8.	Unknown	−	16.1	599.27 *	553.20 391.06 373.13 361.06	−	+	+	−
9.	Olivil-di-*O*-glucoside	275	16.8	745.63 *	699.22 537.17 375.10	Lignans	+	+	[12,14]
10.	5-*O*-caffeoylquinic acid	325	17.9	353.16	190.93	Caffeic acid derivatives	+	+	[12,14]
11.	4-*O*-caffeoylquinic acid	325	18.4	353.41	190.70 178.50 172.75	Caffeic acid derivatives	+	+	[12,14]
12.	Geniposide	225	20.0	433.61	387.07 224.82 122.84	Irydoids	+	−	[12,15]
13.	Unknown	200	20.7	461.40 *	415.11 265.28	−	+	+	−
14.	Olivil-*O*-glucoside (I)	270	21.0	583.30 *	537.19 375.09 357.06	Lignans	+	+	[12]
15.	Coniferin	−	21.9	387.52 *	340.91 284.94 206.78 178.72	Phenolics	+	+	[16,17]
16.	Hydroxypinoresinol di-*O*-glucoside	270	22.3	743.29 *	697.29 535.14 373.19	Lignans	+	+	[12,14]
17.	Olivil-*O*-glucoside (II)	270	22.8	583.29 *	537.16 375.08 194.77	Lignans	+	+	[12,14]
18.	Genipin	200	24.1	225.10	207.08 122.79	Irydoids	+	+	[12,14]
19.	Pinoresinol di-*O*-glucoside (I)	276	25.5	727.21 *	681.22 519.19 357.20	Lignans	+	+	[12,18]
20.	Pinoresinol di-*O*-glucoside (II)	276	26.4	727.37 *	681.30 519.09 357.13 341.08	Lignans	+	+	[12,18]
21.	Citrusin B	275	27.0	613.30 *	567.30 405.12 357.11 208.85	Lignans	+	+	[19]
22.	Syringaresinol-di-*O*- glucoside	275	27.7	787.75 *	741.33 579.30 417.14	Lignans	−	+	[12,14]
23.	Hydroxypinoresinol-*O*-glucoside	275	28.4	581.30 *	535.14 373.07 343.09 313.31	Lignans	+	+	[12]
24.	Olivil	272	29.5	375.64	357.04 327.08 194.72	Lignans	+	+	[14,15]
25.	Unknown	273	30.4	565.36	339.06 327.07	−	+	+	
26.	Eucomoside B	203	31.1	520.29	355.03 233.83 190.75	Irydoids	+	−	[12,14]
27.	Pinoresinol-*O*-glucoside (I)	275	33.4	519.38	357.34	Lignans	+	+	[12,15]
28.	Pinoresinol-*O*-glucoside (II)	275	33.9	565.29 *	519.14	Lignans	+	+	[12,15]
29.	Syringaresinol-*O*-glucoside	275	34.3	579.33	417.11	Lignans	+	+	[12,14]
30.	Unknown	203	34.7	419.39 *	373.06	−	−	+	−
31.	Unknown	343	35.5	563.25	503.08 337.06	−	+	+	−
32.	Dicaffeoylquinic acid	312	37.7	515.34	353.01	Caffeic acid derivatives	+	−	[14,15]
33.	Pinoresinol vanillic acid ether diglucoside	275	38.6	831.37	669.23 519.39 343.57 311.01	Lignans	+	+	[12,14]
34.	Unknown	270	44.3	583.37	535.16 369.08 357.28	−	+	+	−
35.	Unknown	345	47.0	337.46	321.98	−	+	+	−
36.	Pinoresinol vanillic acid ether glucoside	−	47.4	699.24	357.13 343.06 310.97	Lignans	+	+	[11,12,14]
37.	Medioresinol-*O*-guaiacylglycerol ether	−	49.2	583.35	535.20 505.17 387.10 357.15	Lignans	+	+	[11,12,14]
38.	Unknown	320	49.7	581.34	533.16 367.08 355.13	−	+	+	−

* occurs as [M + COOH]^−^.

### 2.2. Content of Leading Compounds in the Tested Extracts

The linearity of two dominant compounds (5-*O*-caffeoylquinic acid and pinoresinol di-*O*-glucoside) was assessed across different concentration levels to establish the calibration curve. The proposed analytical method demonstrated strong linearity for all tested compounds, with correlation coefficient (R²) values exceeding 0.99 (Table 2). Based on the calibration curve, the concentrations of individual compounds in the examined extract samples were determined. The obtained results and mean concentrations of main compounds (mg/g ± SD) are summarized in Table 2.

### 2.3. Effect of EC Extracts on the ROS Release of f-MLP-Stimulated Neutrophils

**Figure 2:**
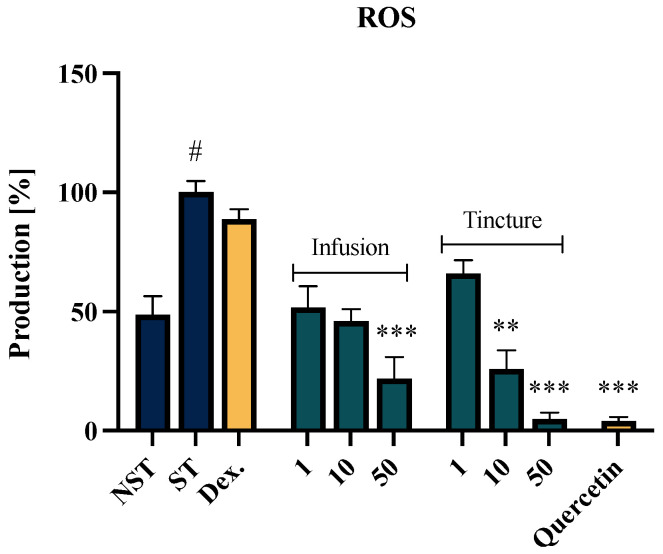
The influence of *Eucommiae cortex* extracts on ROS release by f-MLP stimulated neutrophils. Data from three separate experiments performed using neutrophils isolated from independent donors assayed in duplicate are expressed as mean ± SEM. Quercetin (40 μM) was used as a positive control. Statistical significance: ** *p* < 0.01, *** *p* < 0.001 vs. stimulated control (ST), # statistically significant (*p* < 0.001) vs. non-stimulated control (NST).

The evaluation of the antioxidant properties of Eucommiae cortex extracts was conducted in vitro using human neutrophil cells stimulated with an f-MLP solution. Both the EC infusion and tincture demonstrated a concentration-dependent and statistically significant inhibitory effect on ROS secretion (Figure 2). The highest antioxidant potential was observed with the tincture at a concentration of 50 μg/mL. The ROS secretion at a level of 5.00 ± 2.7% following the treatment was comparable to the activity of the reference compound, quercetin (4.18 ± 1.6%). For the tested infusion, the highest activity was noted at a concentration of 50 μg/mL, which inhibited ROS secretion to 21.8 ± 9.2%, compared to the f-MLP stimulated control (100% secretion). The lowest concentration used (1 µg/mL) showed no statistically significant differences compared to the ST control.

### 2.4. Effects of EC Extracts and Leading Compounds on the Proinflammatory Functions of LPS-Stimulated Neutrophils

The anti-inflammatory potential of the tested extracts, prepared from the investigated plant material, was evaluated using an in vitro neutrophil cell model. The effect of the extract on cell viability was assessed via the LDH assay, and it was found that the tested samples at the evaluated concentrations (50 µg/mL) did not adversely impact the viability of the isolated cells (see Supplementary Materials, Figure S1).

**Figure 3:**
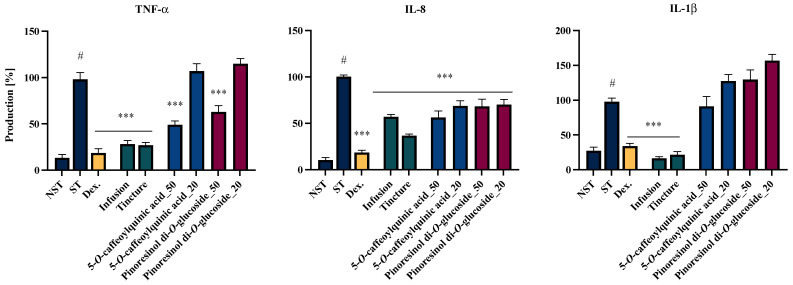
The influences of the infusion, the tincture (50 μg/mL) and the leading compounds (20–50 μM) of the *Eucommiae cortex* on the release of TNF-α, IL-8 and IL-1β by LPS-stimulated neutrophils. Non-stimulated control (NST), LPS-stimulated control (ST), and dexamethasone (Dex.) (20 μM) cells were used as positive controls. Data are expressed as the mean ± SEM of three separate experiments. Statistical significance: *** *p* < 0.001 vs. stimulated control, # statistically significant (*p* < 0.001) vs. non-stimulated control.

Notably, the inhibitory activity against TNF-α and IL-1β secretion was markedly more effective at 50 μg/mL for extracts than the activity observed for IL-8 secretion (Figure 3). Both the infusion and the tincture significantly reduced TNF-α secretion to levels of 28.2 ± 3.8% and 26.9 ± 3.0%, respectively. In the case of IL-1β, the inhibition levels achieved by the infusion and tincture were 16.6 ± 2.2% and 21.6 ± 4.4%, respectively, which are lower than those obtained with the positive control with dexamethasone (33.0 ± 4.2%). When evaluating the impact of the natural product at a concentration of 50 µg/mL on IL-8 secretion, the tincture exhibited a statistically significant inhibitory activity (36.7 ± 1.9%) compared to the tested infusion (57.0 ± 2.5%) (*** *p* <0.001).

To assess whether the leading compounds in the infusion and ethanolic extract are responsible for the activity of the tested formulations, we also evaluated the activities of 5-*O*-caffeoylquinic acid (compound 10) and pinoresinol di-*O*-glucoside (compound 19) in a neutrophil model (Figure 3).

Two analyzed compounds demonstrated inhibitory activity against TNF-α and IL-8 secretion. 5-*O*-caffeoylquinic acid, as well as pinoreninol di-*O*-glucoside, significantly suppressed TNF-α secretion at 50 µM, with inhibition levels of 48.9 ± 4.1%, 39.3 ± 8.0%, and 52.0 ± 6.1%, respectively. Moreover, both compounds significantly reduced IL-8 secretion at both tested concentrations. Notably, 5-*O*-caffeoylquinic acid at a concentration of 50 µM exhibited the highest inhibitory efficacy, decreasing IL-8 release to 56.15 ± 7.9% relative to the LPS-stimulated control (100% secretion). Despite the observed trends, none of the analyzed compounds exhibited statistically significant inhibitory activity against IL-1β secretion.

### 2.5. Effect of EC Extracts and Compounds on the Proinflammatory Function of LPS-Stimulated PMBCs Monocytes/Macrophages

**Figure 4:**
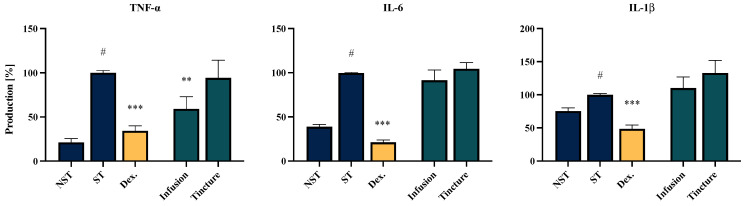
The influence of the infusion and tincture (50 μg/mL) of *Eucommiae cortex* on the release of TNF-α, IL-6 and IL-1β by LPS-stimulated PBMCs monocytes/macrophages. Non-stimulated control (NST), LPS-stimulated control (ST) and dexamethasone (Dex.) (20 μM) cells were used as a positive control. Data are expressed as the mean ± SEM of three separate experiments. Statistical significance: ** *p* < 0.01, *** *p* < 0.001 vs. stimulated control, # statistically significant (*p* < 0.001) vs. non-stimulated control (NST).

Another model used to evaluate the anti-inflammatory potential of *EC* extracts and their main constituents involved PBMC monocytes/macrophages isolated from healthy human donors (Figure 4). Similar to the results obtained with neutrophils, the PBMC monocytes/macrophages showed that the infusion exhibited the highest inhibitory effect on TNF-α secretion, achieving a reduction to 59.1 ± 13.8% compared to the LPS-stimulated control (100% secretion). The effects of the extracts on IL-6 and IL-1β production did not reach statistical significance. None of the tested extracts showed toxicity towards PBMCs/monocytes/macrophages (see Supplementary Materials, Figure S2).

### 2.6. Effect of EC Extracts and Compounds on the Proinflammatory Function of LPS-LPS-Stimulated THP-1-Derived Macrophages

**Figure 5:**
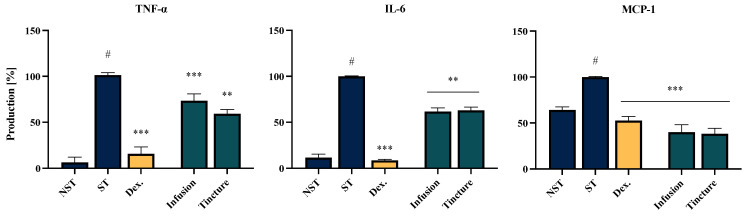
The influence of the infusion and tincture (50 μg/mL) of *Eucommiae cortex* on the release of TNF-α, IL-6 and MCP-1 by LPS-stimulated THP-1-derived macrophages. Non-stimulated control (NST), LPS-stimulated control (ST), and dexamethasone (Dex.) (20 μM) cells were used as a positive control. Data are expressed as the mean ± SEM of three separate experiments. Statistical significance: ** *p* < 0.01, *** *p* < 0.001 vs. stimulated control, # statistically significant (*p* < 0.001) vs. non-stimulated control (NST).

The third model included in the bioassay involved macrophages differentiated from the THP-1 monocytic cell line. The findings from this cellular model partially confirm those observed in the PBMC-derived monocyte/macrophage model, with one notable difference—both tested extracts demonstrated a statistically significant inhibitory effect on IL-6 production. Specifically, the infusion and tincture reduced IL-6 production to 61.7 ± 4.0% and 63.0 ± 3.5%, respectively (without cytotoxic effect, see Supplementary Materials, Figure S3). Additionally, both extracts exhibited inhibitory activity against MCP-1 secretion, with reductions to 40.0 ± 7.9% and 38.3 ± 6.0%, respectively (Figure 5).

## 3. Discussion

*Eucommia ulmoides* Oliver (*EU*), a species within the family Eucommiaceae, holds a prominent position in traditional herbal medicine across several Asian countries, including Korea, Japan, and China. Various *E. ulmoides* components, such as bark, leaves, stems, and flowers, have traditionally been used as a non-toxic and relatively safe natural ingredient, either in monotherapy or as part of complex formulations in traditional medicinal practices.

Ethnopharmacological reports and a long tradition of use in Traditional Chinese Medicine (TCM) have shown the significant biological activity of *Eucommiae cortex* (*EC*), noting its possible application in auxiliary therapies of hyperuricemia, hyperlipidemia, obesity and diabetes. Notably, *EC* is the active component of the Chinese patented pharmaceutical product QuanDuzhong Capsules, which is officially listed in the 2020 edition of the Chinese Pharmacopoeia as a primary therapeutic agent for hypertension [20]. Additionally, in vivo investigations have corroborated the anti-osteoporotic properties of *EC* and its extracts. Traditionally, *EC* has been widely employed in China to alleviate musculoskeletal disorders, particularly for the management of rheumatoid arthritis. Furthermore, recent scientific studies have elucidated the neuroprotective and cardioprotective effects of this plant material [21,22,23].

A common factor significantly contributing to the pathomechanism of the aforementioned conditions, including a group of non-communicable diseases (NCDs) such as type II diabetes, arthritis, atherosclerosis, and the constellation of factors associated with Metabolic syndrome—dyslipidemia, hyperglycemia, insulin resistance, and even osteoporosis—is chronic inflammation, characterized by a progressive increase in severity over time [24,25,26]. The inflammatory response is initiated as an adaptive mechanism whereby immune cells are activated to eliminate harmful agents and promote tissue repair. However, excessive or dysregulated inflammation can lead to tissue damage, while prolonged inflammatory processes contribute to the development and progression of chronic inflammatory diseases.

*Eucommia ulmoides* (*EU*) has demonstrated notable anti-inflammatory properties in both clinical applications and experimental conditions, particularly in osteoarthritis and rheumatoid arthritis models. However, the mechanisms underlying the pharmacological effects of the raw material remain insufficiently elucidated.

A substantial body of existing research has concentrated on evaluating the anti-inflammatory and antioxidant properties of *Eucommiae* leaves [27,28,29,30,31], flowers [32,33,34] and seeds [35], as well as their specific constituents. The emphasis on these specific parts of the plant arises primarily from their abundant availability in Asian regions and the great diversity of their phytochemical profiles.

Due to the relatively limited number of reports on the bark of *Eucommia ulmoides*, our study aimed to evaluate the phytochemical profiles and biological activities of two formulations prepared from this raw material. During the research, we assessed the phytochemical composition and quantitatively identified the major compounds in both an infusion and a 60% ethanolic extract. Subsequently, we evaluated the potential anti-inflammatory activities of these samples in three cellular models—neutrophils, peripheral blood mononuclear cells (PBMCs) isolated from human peripheral blood, and the human monocytic cell line THP-1. Due to the significant activity of the extracts in human neutrophils, the assessment of the activity of the dominant compounds in the studied extracts was also conducted using this model (Figure 3). A complementary assessment of the tested extracts in a model based on unmodified human immune cells may provide a relatively accurate representation of the raw material’s potential effects on the human immune system.

The highest anti-inflammatory potential of *EC* extracts was observed in the model of neutrophils isolated from human peripheral blood. The effective regulation of systemic and local neutrophil activation is crucial for mitigating infections and resolving inflammation, thereby preventing progression to tissue damage or the escalation of widespread inflammatory responses. Both the *EC* infusion and tincture significantly suppressed the secretion of key neutrophils’ inflammatory mediators. The most pronounced effects were observed in the case of IL-1β and TNF-α secretion, with levels reduced to those comparable to the positive control, dexamethasone. IL-1β secretion was notably minimized to 16.6 ± 2.2% and 21.6 ± 4.4% for the infusion and tincture, respectively. This reduction is particularly noteworthy given the critical role of IL-1β in regulating neutrophil recruitment through the upregulation of adhesion molecules on endothelial cells and the localized production of IL-8 chemokine [36]. In addition, both the *EC* infusion and tincture inhibited IL-8 secretion in the neutrophil model, with reductions to 57.0 ± 2.45% and 36.7 ± 1.89%, respectively. This finding holds particular significance due to IL-8’s involvement in promoting chemotaxis and NETosis in human peripheral blood neutrophils [37]. These results underscore the therapeutic potential of *EC* extracts in modulating neutrophil-mediated inflammatory responses.

The observed differential ability of *EC* extracts to inhibit IL-1β secretion in neutrophils but not in PBMC-derived monocytes/macrophages may be attributed to cell-specific mechanisms. In neutrophils, IL-1β secretion is closely associated with TLR4 activation and oxidative stress-related pathways, which appear to be more responsive to modulation by the bioactive compounds present in the *EC* extracts. Conversely, IL-1β production in PBMC-derived monocytes/macrophages typically requires dual signaling involving the combined activation of TLR4 and the NLRP3 inflammasome. This suggests that the different mechanisms of IL-1β production in these cell types may explain the variability in response to *EC* extracts [38,39].

A notable finding from the present study is the impact of *EC* on the modulation of mononuclear cell functions, which are involved in subsequent stages of the development, maintenance and resolution of inflammation [40]. In the THP-1-derived macrophage model, *EC* extracts significantly suppressed the secretion of all investigated pro-inflammatory mediators, including TNF-α, IL-6, and MCP-1. For TNF-α, the *EC* infusion showed greater efficacy in the peripheral blood mononuclear cell (PBMC) model, reducing its secretion to 59.1 ± 13.8%. In contrast, the tincture exhibited superior activity in the THP-1-derived macrophage model, reducing TNF-α secretion to 31.2 ± 5.54%.

Monocyte chemoattractant protein-1 (MCP-1/CCL2) is a key chemokine regulating the migration and infiltration of monocytes/macrophages across the vascular endothelium. The inhibition of MCP-1 secretion may play a critical role in shaping the inflammatory response and promoting the restoration of homeostasis [41]. In our study, MCP-1 secretion was reduced to 40.1 ± 7.9% and 38.3 ± 6.0% for the infusion and tincture, respectively. These values indicate higher activity compared to the positive control with dexamethasone (52.6 ± 4.4%). Furthermore, no significant difference was observed between the infusion and tincture in terms of the effect on IL-6 secretion, with an average secretion level of 62%.

To contextualize our findings, which underscore the pronounced anti-inflammatory potential of *Eucommia ulmoides* bark (*EC*), within the framework of existing research, it is pertinent to highlight that the majority of prior studies rely on animal cell models, which do not fully reflect the effects within the human organism. To the best of our knowledge, our study represents one of the first to elucidate the modulatory effects of *EC* using human immune in vitro models, specifically assessing its influence on the production of inflammatory mediators and reactive oxygen species (ROS).

Nonetheless, it is noteworthy that the findings of this study are consistent with previously reported data, including those utilizing macrophage models of non-human origin. For instance, Kim et al. demonstrated the anti-inflammatory potential of *EC*, reporting the significant inhibition of TNF-α and IL-6 secretion in LPS-stimulated peritoneal mouse macrophages [42]. Similarly, Kwon et al. investigated the anti-inflammatory properties of *EU* bark extract in activated BV-2 microglial cells, highlighting the suppression of NF-κB signaling and the upregulation of heme oxygenase-1 (HO-1) via the Nrf2 pathway. Similarly, Koh W. et al. identified the inhibitory effect of *EC* decoction on the production of inflammatory mediators primarily through interference with the Myd88-dependent (MAPK pathways suppression) or the Myd88-independent pathway (suppress IFN-β and STAT pathway) of TLR-4 signaling. Additionally, the *EC* extract was shown to enact a dose-dependent downregulation of inducible nitric oxide synthase (iNOS), cyclooxygenase-2 (COX-2), TNF-α, IL-1β, and components of the PI3K/Akt/mTOR signaling cascade [43].

Free radicals, naturally produced during human metabolism, play critical roles in immune defense and cellular signaling. However, aging and physiological degradation disrupt the balance between oxidative and antioxidant mechanisms, leading to the accumulation of excess free radicals. This imbalance causes cellular damage, inflammatory responses, and the production of oxidative intermediates, which are key contributors to aging and the development of chronic diseases. In the study by Xu Z. et al., evaluating the antioxidant activity of ethanolic extracts prepared from different parts of the *EU*, the highest antioxidant activity was observed for *EU* leaves, bark and seeds extracts, respectively [44]. Despite the similar antioxidant profiles of *Eucommiae* bark and leaves, notable differences in their phytochemical compositions appear to significantly influence the observed biological effects of these two plant materials. In contrast to the leaves, *EC* lacks certain flavonol derivatives, such as rutin (quercetin 3-*O*-rhamnoglucoside), hyperoside (quercetin 3-*O*-galactoside), and astragalin (kaempferol 3-*O*-glucoside) [45]. Despite this, due to the presence of caffeic acid derivatives and other polyphenolic compounds, *EC* caught our attention for its potential antioxidant activity. In this context, the findings of our study serve as a valuable complement to those of Luo J. et al., who evaluated the potential effect of *EC* on DPPH, Fe^2+^-chelating ability and lipid peroxidation. Notably, our study revealed a concentration-dependent ability to inhibit ROS production in fMLP-stimulated neutrophils, even at low concentrations of the tested *EC* infusion or tincture (10 or 50 µg/mL). In contrast to previous studies that evaluated extract activity at much higher concentrations (ranging from 0.02 to 2.5 mg/mL), our findings highlight the effectiveness of *EC* infusions and tinctures, reinforcing their antioxidant (in the context of both scavenging and inhibiting the production of free radicals) potential for practical therapeutic use [46].

A crucial aspect of research on plant-based products involves addressing their actions and correlating the observed effects with specific constituents of the raw material or its metabolites. Synergistic interactions among individual components within a formulation are frequently observed, particularly in natural products composed of diverse chemical compounds. Such synergy can result in additive or even potentiated effects across various chemical groups, thereby enhancing the overall biological activity—for example, anti-inflammatory effects.

Quantitative analyses have identified lignans, such as pinoresinol, olivil, and syringaresinol derivatives, as major phytochemicals in *EC* alongside iridoids (such as aucubin geniposide) and fenolic acid derivatives (such as 5-*O*-caffeoylquinic acid) [45]. These findings align closely with the phytochemical profile described in the present study (Table 1). Specifically, the infusion exhibited a higher concentration of iridoid compounds compared to the tincture, while the levels of lignans were similar in both extracts.

Interestingly, according to the analysis conducted, the content of the aforementioned compounds was relatively higher in the infusion compared to the ethanolic extract (Table 2). These compositional differences are likely to contribute to the observed variations in the biological effects of the two analyzed samples; for example, in the PBMC monocyte/macrophage cell model.

Among the natural lignans identified in the tested samples, the presence of pinoresinol diglucoside was confirmed, which, according to the Chinese Pharmacopoeia (2020 edition), is recognized as a quality control marker for *EC* [47]. Additionally, pinoresinol monoglucoside and its aglycone, as well as hydroxypinoresinol, olivil, syringaresinol, and their glycoside derivatives, were identified in both extracts. The evidence from the literature indicates that these lignan structures possess anti-inflammatory properties, suggesting their involvement in the observed anti-inflammatory and antioxidant effects of *EC* [48]. This is further supported by our analysis of the anti-inflammatory activity of individual compounds, including 5-*O*-caffeoylquinic acid and pinoresinol di-*O*-glucoside, which, at the higher tested concentration (50 µM), significantly reduced the production of TNF-α and IL-8 in a statistically significant manner. Interestingly, none of the analyzed compounds demonstrated a significant inhibitory effect on IL-1β secretion, which may suggest the involvement of other structural components in reducing this mediator’s secretion.

Although the content of compounds from the iridoid group in the analyzed extracts was relatively low, available reports highlight the involvement of this group in the anti-inflammatory activity of *EC* [49,50]. Aucubin received special attention, demonstrating its inhibitory effect on LPS-induced inflammation and apoptosis in a mouse model of cardiac dysfunction [51] or acute pulmonary injury [52]. Similarly, geniposide was found to inhibit NLRP3 inflammasome activation via autophagy in BV-2 microglial cells exposed to oxygen–glucose deprivation/reoxygenation, and to ameliorate dextran sulfate-induced colitis in mice via the Nrf2/HO-1/NF-κB pathway [53]. In turn, Sun J. et al. demonstrated that geniposidic acid extracted from *EU* could alleviate osteoarthritis progression by inhibiting inflammation and chondrocyte ferroptosis [49]. However, the effects of these compounds in models of human immune cells remain unexplored.

Considering the critical role of the inflammatory component and oxidative action in the development of inflammatory-based diseases, the results presented in this paper regarding the activity of *EC* extracts in physiological macrophages and neutrophils may serve as an important basis for expanding research on the use of this raw material in the prevention of various chronic inflammatory diseases that result from the excessive activation of leukocytes. Considering the results obtained for the dominant compounds in the analyzed extracts, specifically 5-*O*-caffeoylquinic acid and pinoresinol di-*O*-glucoside, it can be hypothesized that these compounds significantly contribute to the observed indirect effects of the raw material on its health-promoting properties.

## 4. Materials and Methods

### 4.1. Chemicals and Reagents Used in the Study

Plant material analysis—Ethanol purchased from Avantor Performance Materials Poland S.A. was used to prepare the alcoholic extract. All solvents used for chromatography were of gradient grade. Distilled water was purified with the Millipore Simplicity UV system (Bedford, MA, USA).

Neutrophils and PBMC monocytes/macrophages isolation, THP-1 cell culture and in vitro investigations—Citric acid, sodium citrate, glucose, dextran, sodium chloride, and potassium chloride were purchased from Sigma-Aldrich Chemie GmbH (Steinheim, Germany). HEPES buffer was purchased from Sigma-Aldrich Chemie GmbH (Steinheim, Germany). RPMI 1640 medium and L-glutamine (Mg^2+^, Ca^2+^)-free phosphate-buffered saline (DPBS) were purchased from Thermo Fisher Scientific (Waltham, MA, USA). Ficoll Hypaque gradient (LSM 1077) and penicillin–streptomycin solution were obtained from PAA, Laboratories GmbH (Pasching, Austria). LPS (from *Escherichia coli* 0111:B4) and DMSO were purchased from Sigma-Aldrich Chemie GmbH (Steinheim, Germany). Human Quantikine ELISA Kits were purchased from the BD Biosciences R&D System (San Jose, CA, USA). Dexamethasone was purchased from Merck (Darmstadt, Germany). Quercetin was purchased from Carl Roth (Karlsruhe, Germany).

### 4.2. Plant Material

The plant material originated from China (Du Zhong/*Eucommiae Cortex*) and was purchased commercially from Planetherbs (ref. TJ20220114039; expiration date November 2024). A voucher specimen of the tested plant material has been deposited in the Plant Collection of the Department of Pharmaceutical Biology at the Medical University of Warsaw (Warsaw, Poland).

### 4.3. Extracts Preparation

The *Eucommiae cortex* dry sample (100 g) was finely ground and crushed. Infusions were prepared by adding boiling water at a ratio of 1:10 (50 g of plant material and 500 mL of water) and left for 15 min under cover. Tinctures were prepared by adding 60% ethanol to the finely ground and crushed plant material (1:5 ratio—50 g of plant material and 250 mL of 60% ethanol) and heating at 95 °C for one hour. After solvent evaporation, the aqueous residues were lyophilized and stored for LC-MS analysis, compound isolation and bioassays.

### 4.4. UHPLC-DAD-ESI-MS/MS Analysis

UHPLC-DAD-ESI-MS/MS analysis was performed on a UHPLC-3000 RS system (Dionex, Germering, Germany) with DAD detection and an AmaZon SL ion trap mass spectrometer with an ESI interface (Bruker Daltonik GmbH, Bremen, Germany). Separation was performed on a Zorbax SB-C18 column (150 × 2.1 mm, 1.9 μm) (Agilent, Santa Clara, CA, USA). The mobile phase consisted of water + 0.1% formic acid (A) and acetonitrile + 0.1% formic acid (B). A gradient elution was applied as follows: 0–60 min, 5–40% B. The LC eluate was introduced into the ESI interface without splitting, and the analysis was performed in both negative and positive ion modes with the following parameters: nebulizer pressure at 40 psi; drying gas flow rate at 9 L/min; nitrogen gas temperature at 300n°C; and a capillary voltage of 4.5 kV. The mass scan range was set from 100 to 2200 *m*/*z*. UV–Vis detection covered the 190–600 nm range. Samples for phytochemical analysis were prepared at a concentration of 5 mg/mL, and the injection volume was 5 µL.

All samples were analyzed using the UHPLC-DAD-ESI-MS/MS method, and the predominant substances in each extract were identified by comparing retention times, spectra (UV, MS, MS/MS) and literature data.

### 4.5. Extract Fractionation and Isolation of Active Compounds

The collected ethanolic extract was further separated using column chromatography. The extract components were adsorbed onto a glass column (43.5 × 5 cm) filled with Diaion HP-20 and subsequently eluted with 0.5 L of water, followed by 20% methanol, 50% methanol, 70% methanol, and 100% methanol, respectively. The methanol from each fraction was evaporated under reduced pressure, and the aqueous residues were lyophilized to obtain five fractions (F1-F5) weighing 1.74 g, 1.05 g, 0.49 g, 0.75 g, and 0.8 g, respectively. All fractions were characterized using the HPLC-DAD-MS/MS method.

From fraction F4 (0.75 g), two compounds were isolated using preparative HPLC with a 0.1% HCOOH in H_2_O (A) and 0.1% HCOOH in MeCN (B) gradient (5:40 → 0:60 min) over 20 min. The compounds that were isolated include the following: 5-*O*-caffeoylquinic acid (12.51 → 12.9 min, 7.7 mg) and pinoresinol di-*O*-glucoside (19.02 → 19.40 min, 6.8 mg) (see Supplementary Materials—Figures S4 and S5). Preparative HPLC was performed with a Shimadzu LC-20AP instrument (Tokyo, Japan) using a Kinetex XB-C18 column (Phenomenex, Torrance, CA, USA, particle size 5.0 µm, 150 × 21.2 mm) at a flow rate of 20.0 mL/min.

NMR spectra for isolated compounds were registered with a Bruker Avance Neo 400 spectrometer (Bruker Biospin, Ettlingen, Germany) resonating at 400.15 MHz for 1 h. All measurements were performed at 295 K. All signals were calibrated at solvent residual signals at 3.31 ppm for 1 h (methanol d4).

### 4.6. Determination of the Content of Predominant Compounds in the Analyzed Extracts

The five-point calibration curve for the 2 major compounds (5-*O*-caffeoylquinic acid and pinoresinol di-*O*-glucoside) shows a linear correlation between compound concentration and peak area. The calibration data indicate the linearity (R^2^ > 0.99) of the detector response for the 2 compounds over the concentration range of 0.5–10 µg/mL. All samples and standards were injected in triplicates.

### 4.7. Preparation of Samples for Bioassay

Tested samples were dissolved in DMSO and then diluted with (Mg^2+^, Ca^2+^)-free PBS buffer in a pH 7.4 solution, reaching a final concentration of 1 mg/mL. Single compounds and positive controls with dexamethasone and quercetin, were dissolved in DMSO (10 mM stock solution) and then diluted with (Mg^2+^, Ca^2+^)-free PBS buffers at pH 7.4 or RPMI 1640 medium. The extracts were tested at a concentration of 50 µg/mL, while the individual compounds were evaluated within a concentration range of 20–50 µM. Positive controls were tested at the concentrations of 20 µM and 40 µM for dexamethasone and quercetin, respectively.

### 4.8. Isolation of Human Neutrophiles and PBMCs Monocytes/Macrophages

The study was conducted according to the principles of the Declaration of Helsinki, with consent granted by the Local Bioethics Committee (no. AKBE/54/2023). Peripheral venous blood was collected from the Warsaw Blood Donation Centre. Male healthy human donors (18–35 years old) declared themselves healthy, non-smokers, and not taking long-term pharmacotherapy.

As previously described, neutrophils were isolated by dextran sedimentation, erythrocyte lysis, and centrifugation in a Ficoll–Hypaque gradient [54]. After the isolation process, neutrophils were suspended in RPMI 1640 medium supplemented with 10% Fetal Bovine Serum (FBS), 10 mM HEPES, 2 mM L-glutamine and penicillin/streptomycin or HBSS buffer, or (Ca^2+^)-free PBS buffers at pH 7.4. Depending on the type of experiment, the cell suspension was transferred to 96-well plates for ROS analysis or 24-well plates for the analysis of cytokines’ release.

PBMC monocytes/macrophages were isolated using a Ficoll–Hypaque gradient, according to the method of Zapolska-Downar et al. [55]. The mononuclear cell band was removed by aspiration, and cells were suspended in RPMI 1640 medium with L-glutamine, antibiotics and autologous serum (20%). To allow the adherence of monocytes/macrophages, the peripheral blood mononuclear cell suspension was placed in 12-well tissue culture plates (2 × 10^6^ per well) and incubated for 2 h under standard conditions (37 °C, 5% CO_2_). After this time, non-adherent cells were removed, and adherent cells were cultivated in the same medium and conditions for 3 days. The medium and autologous serum was replaced every 48 h.

### 4.9. Human Monocytic Cell Line (THP-1) Cell Culture

Experiments were performed on a human monocyte-like cell line (THP-1) derived from a patient with leukemia obtained from the ATCC bank (ATCC #TIB-202). Cell suspensions were cultured in RPMI 1640 supplemented with 10% FBS at 37 °C and 5% CO_2_. After seeding (4 × 10^5^ cells/well in a 24-well plate for ELISA assay, or 5 × 10^4^ cells/well in a 96-well plate for MTT assay), cells were differentiated into macrophages by incubation with 25 ng/mL phorbol 12-myristate 13-acetate (PMA) in cell culture medium for 48 h at 37 °C and 5% CO_2_, followed by 24 h of incubation of the cells without differentiation agent.

### 4.10. Cytotoxicity and Metabolic Activity Determination

The cytotoxicity of the tested extracts was determined by use of a lactate dehydrogenase (LDH) release assay kit. Neutrophils and PBMC monocytes/macrophages were exposed to the tested extracts (or appropriate controls), with or without stimulation with LPS for 24 h. After the incubation time, supernatants were harvested, and the LDH level was measured according to the protocol. Absorbance was read at 490 nm using a microplate reader. TRITON X-100 (2%) was used as a positive control.

THP-1-derived macrophages metabolic activity was assessed through a 3-(4,5-dimethylthiazol-2-yl)-2,5-diphenyltetrazolium bromide (MTT) assay. PMA-differentiated THP-1 cells, previously plated at a density of 5 × 10^4^ cells/well in a 96-well plate, were exposed for 24 h to tested samples in a complete medium. Before the assay, each well was washed with Dulbecco’s phosphate-buffered saline (DPBS) to remove the extracellular particles and eventual cell debris and treated with MTT (0.5 mg/mL) in RPMI medium for 4 h under standard conditions. Then, the MTT reagent was discarded, and dimethyl sulfoxide (DMSO) was added to each well (200 µL) to solubilize formazan crystals. The absorbance of each well was measured using a microplate reader at 570 and 630 nm. TRITON X-100 (0.1%) was used as a positive control.

### 4.11. ROS Secretion by Human Neutrophils

The production of reactive oxygen species (ROS) was evaluated using the luminol-dependent chemiluminescence method following stimulation with N-formyl-l-methionyl-l-leucyl-l-phenylalanine (f-MLP). A 70 μL cell suspension (2 × 10^5^) in Ca^2+^-free HBSS, combined with the tested extracts, was incubated with 50 μL of luminol solution (100 μM). ROS generation was triggered by adding 30 μL of f-MLP (0.1 μg/mL). Chemiluminescence measurements were taken every 2 min over 40 min using a 96-well microplate reader (Synergy 4, BioTek, Winooski, VT, USA). The ROS secretion was quantified as a percentage relative to stimulated control samples. Quercetin (40 μM) was used as a positive control in the experiments. All chemical reagents were obtained from Sigma-Aldrich (St. Louis, MO, USA).

### 4.12. TNF-α, IL-6, IL8, MCP-1 and IL-1β Secretion

Neutrophils (2 × 10^6^ cells/mL), PBMCs monocytes/macrophages (4 × 10^5^ cells/mL) and THP-1 cells (4 × 10^5^ cells/mL) were plated in 24- or 12-well plates and cultivated for 24 h under standard treatment conditions. Subsequently, the cells were treated with tested extracts (50 μmol/L) or single compounds (20–50 μM), followed by stimulation with 100 ng/mL LPS (from *Escherichia coli* O111:B4). After 24 h, the collected supernatants were centrifuged, and the cytokines’ release was determined by use of ELISA assay kits following the manufacturer’s instructions using a microplate reader. The effect on cytokine production was determined through the percentage of released cytokines relative to the LPS-stimulated control without the tested samples. Dexamethasone (20 µM) was used as a positive control.

### 4.13. Statistics and Data Analysis

The results are expressed as mean ± SEM for three independent experiments performed at least in triplicate. One-way analysis of variance (ANOVA), HSD Tukey test, and Dunnett’s test were applied to evaluate the statistical significance of the mean values, with * *p* < 0.05, ** *p* < 0.01 and *** *p*< 0.001 regarded as statistically significant. GraphPad Prism 10 was used for all analyses and graphs.

## 5. Conclusions

The HPLC-DAD-MS phytochemical analysis of *Eucomiae cortex* infusion and tincture and the identification of its primary constituents was combined with the bioassay potential of tested samples. The anti-inflammatory activity of these extracts was assessed through three in vitro cellular models. These findings suggest the potential of *Eucommiae cortex* to be promoted within European regions and applied in traditional phytotherapy. Additionally, the relatively easy accessibility and low cost of *Eucommiae cortex* in trade suggest its potential as a plant material for the isolation of anti-inflammatory constituents.

## Figures and Tables

**Table 2:** Linearity and calibration curves of 2 leading compounds from *E. cortex* extract.

No.	Analytes	Calibration Curves ^a^	R^2^	Mean Concentration (mg/g)	
				Infusion	Ethanolic Extract
1.	5-*O*-caffeoylquinic	y = 5161.5x − 94.68	0.99	12.0 ± 2.6	5.2 ± 2.8
2.	Pinoresinol di-*O*-glucoside	y = 1781x − 2.87	1.00	23.6 ± 0.6	11.7 ± 0.2

^a^ y and x indicate the peak area and concentration of each analyte, respectively, using the standard solution.

## Data Availability

The original contributions presented in this study are included in the article/Supplementary Materials. Further inquiries can be directed to the corresponding authors.

## Supplementary Materials

The following supporting information can be downloaded at: www.mdpi.com/article/10.3390/molecules30061364/s1. Figure S1: The influence of tested extracts (50 μg/mL) and single compounds (20–50 μM) on LDH secretion by LPS-stimulated neutrophils. Figure S2: The influence of the infusion and tincture of *Eucommiae cortex*, tested at 50 μg/mL, on LDH secretion by LPS-stimulated PBMCs monocytes/macrophages. Figure S3: Cytotoxicity of infusion and tincture of *Eucommiae cortex*, tested at 50 μg/mL in MTT assay, in THP-1-derived macrophages. Figure S4: LC-MS chromatogram of 5-*O*-caffeoylquinic acid recorded at 280 nm. Figure S5: LC-MS chromatogram of pinoresinol di-*O*-glucoside recorded at 280 nm.

## Abbreviations

The following abbreviations are used in this manuscript:

fMLP	N Formylmethionyl-leucyl-phenylalanine
LDH	Lactate dehydrogenase
LPS	Lipopolysaccharide
ROS	Reactive oxygen species
TCM	Traditional Chinese Medicine
