# Supplementary material for: Eucommiae cortex Comprehensive Phytochemical Analysis Connected with Its In Vitro Anti-Inflammatory Activity in Human Immune Cells
Source: Molecules. 2025 Mar 18;30(6):1364. doi: 10.3390/molecules30061364 (PMC11944357; doi:10.3390/molecules30061364)
Supplement: Supplementary file 1 [file molecules-30-01364-s001.zip › molecules-3447140-supplementary.pdf]

Supplementary Materials

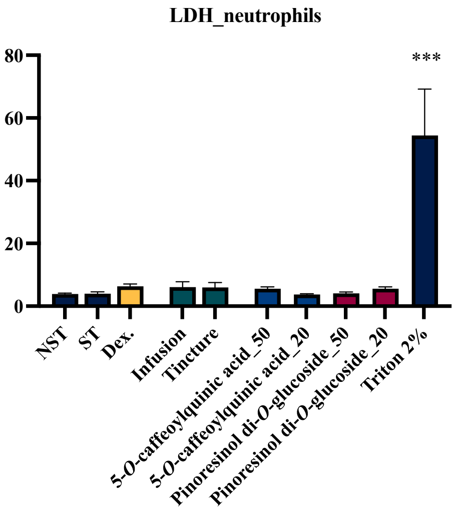

Figure S1. The influence of tested extracts (50 μg/mL) and single compounds (20–50 μM) on LDH secretion by LPS-stimulated neutrophils. Data from three separate experiments performed using neutrophils isolated from independent donors assayed in duplicate are expressed as mean ± SEM. Triton 2% was used as a positive control. Statistical significance: *** $p < 0.001$ vs. stimulated control (ST).

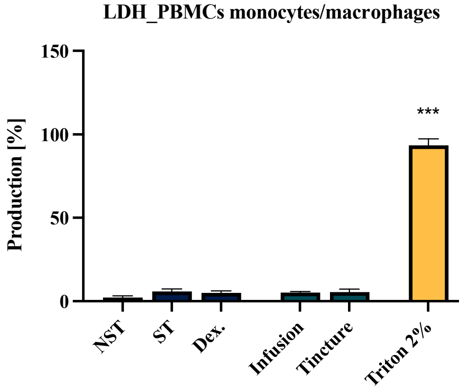

Figure S2. The influence of tested extracts (50 μg/mL) on LDH secretion by LPS-stimulated PBMCs monocytes/macrophages. Data from three separate experiments performed using neutrophils isolated from independent donors assayed in duplicate are expressed as mean ± SEM. Triton 2% was used as a positive control. Statistical significance: *** $p < 0.001$ vs. stimulated control (ST).

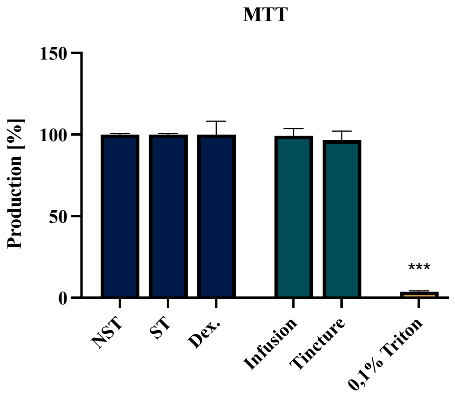

Figure S3. The influence of tested extracts (50 μg/mL), on the cell viability of THP-1-derived macrophages evaluated by MTT assay. Data from three separate experiments assayed in duplicate are expressed as mean ± SEM. Triton 0.1% was used as a positive control. Statistical significance: *** $p < 0.001$ vs. stimulated control (ST).

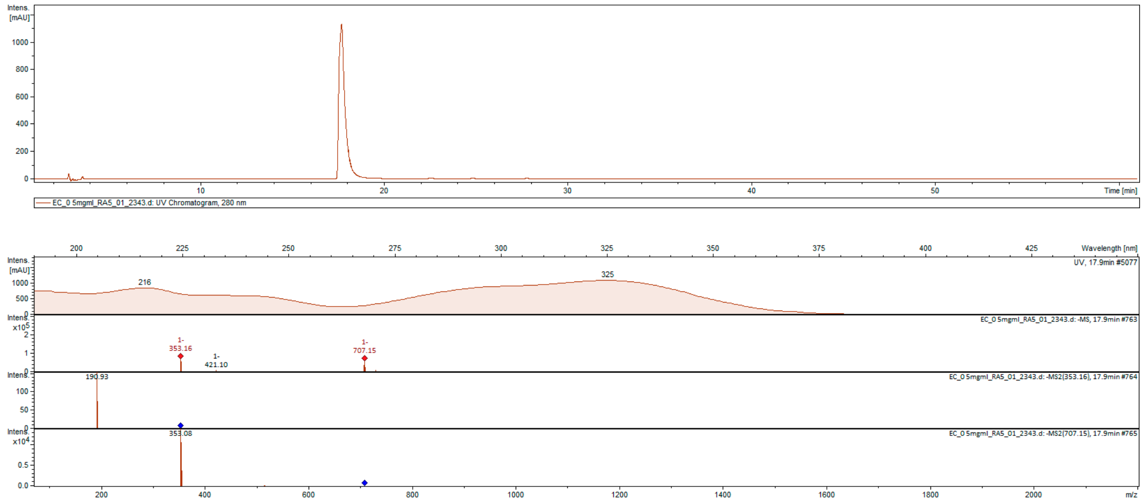

Figure S4. LC-MS chromatogram of 5-*O*-caffeoylquinic acid recorded at 280 nm.

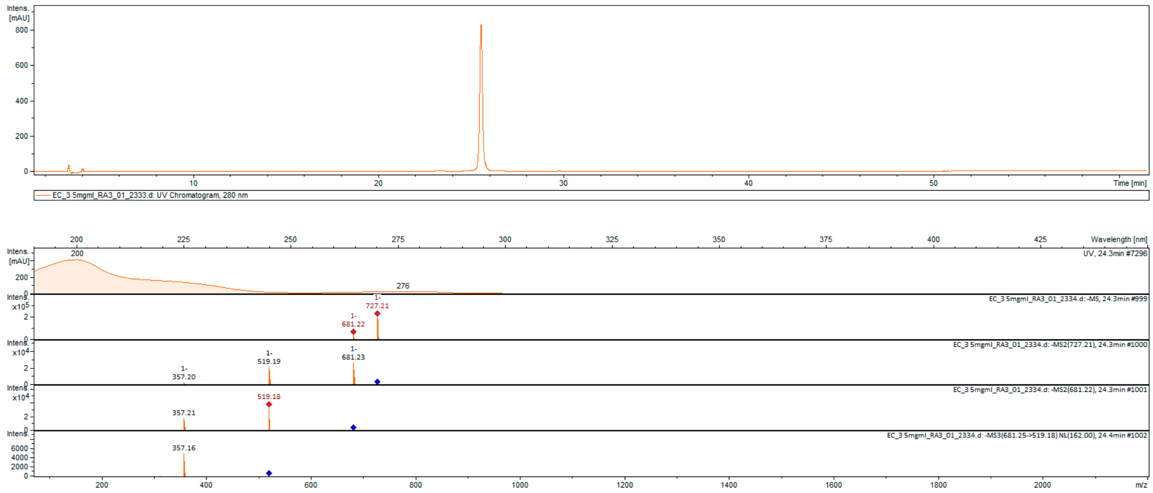

Figure S5. LC-MS chromatogram of pinoresinol di-*O*-glucoside acid recorded at 280 nm.
